# Rhombomere-specific analysis reveals the repertoire of genetic cues expressed across the developing hindbrain

**DOI:** 10.1186/1749-8104-4-6

**Published:** 2009-02-10

**Authors:** David Chambers, Leigh Jane Wilson, Fabienne Alfonsi, Ewan Hunter, Uma Saxena, Eric Blanc, Andrew Lumsden

**Affiliations:** 1 MRC Centre for Developmental Neurobiology, King's College London, Guy's Campus, London, SE1 1UL, UK; 2 Infogen Bioinformatics Ltd, 83 South Middleton, Uphall, West Lothian, EH52 5GA, UK

## Abstract

**Background:**

The Hox family of homeodomain transcription factors comprises pivotal regulators of cell specification and identity during animal development. However, despite their well-defined roles in the establishment of anteroposterior pattern and considerable research into their mechanism of action, relatively few target genes have been identified in the downstream regulatory network. We have sought to investigate this issue, focussing on the developing hindbrain and the cranial motor neurons that arise from this region. The reiterated anteroposterior compartments of the developing hindbrain (rhombomeres (r)) are normally patterned by the combinatorial action of distinct Hox genes. Alteration in the normal pattern of Hox cues in this region results in a transformation of cellular identity to match the remaining Hox profile, similar to that observed in *Drosophila *homeotic transformations.

**Results:**

To define the repertoire of genes regulated in each rhombomere, we have analysed the transcriptome of each rhombomere from wild-type mouse embryos and not those where pattern is perturbed by gain or loss of Hox gene function. Using microarray and bioinformatic methodologies in conjunction with other confirmatory techniques, we report here a detailed and comprehensive set of potential Hox target genes in r2, r3, r4 and r5. We have demonstrated that the data produced are both fully reflective and predictive of rhombomere identity and, thus, may represent some the of Hox targets. These data have been interrogated to generate a list of candidate genes whose function may contribute to the generation of neuronal subtypes characteristic of each rhombomere. Interestingly, the data can also be classified into genetic motifs that are predicted by the specific combinations of Hox genes and other regulators of hindbrain anteroposterior identity. The sets of genes described in each or combinations of rhombomeres span a wide functional range and suggest that the Hox genes, as well as other regulatory inputs, exert their influence across the full spectrum of molecular machinery.

**Conclusion:**

We have performed a systematic survey of the transcriptional status of individual segments of the developing mouse hindbrain and identified hundreds of previously undescribed genes expressed in this region. The functional range of the potential candidate effectors or upstream modulators of Hox activity suggest multiple unexplored mechanisms. In particular, we present evidence of a potential new retinoic acid signalling system in ventral r4 and propose a model for the refinement of identity in this region. Furthermore, the rhombomeres demonstrate a molecular relationship to each other that is consistent with known observations about neurogenesis in the hindbrain. These findings give the first genome-wide insight into the complexity of gene expression during patterning of the developing hindbrain.

## Background

Appropriate specification of body axes during development requires the precise control of a co-ordinated system of positional cues. How this is achieved by the diverse members of the animal kingdom has been the subject of much research and more speculation for many years. Significant progress came from the discovery of mutations in the Hox family of homeobox genes in *Drosophila*, where each Hox gene is expressed in a discrete domain along the anteroposterior (AP) axis, its activity confering a distinct identity on that region. Loss of function of a particular Hox gene leads to a predictable change in identity of the region in which the Hox gene was expressed. Defined as 'homeotic transformation', these findings clearly identified Hox genes as key regulators of positional identity [1-3].

Hox genes code for transcriptional regulators with a highly conserved 180 base-pair homeobox sequence that encodes a 60 amino acid DNA binding domain known as the homeodomain [4-6]. In *Drosophila*, the Hox genes are located in a single complex (HOM-C) that comprises two clusters of genes, the Antennapedia (ANT-C) and Bithorax (BX-C) complexes [2,7]. To further underline their pivotal role in directing body patterning, Hox genes have been discovered in virtually all metazoans studied to date [8]. In the mouse, the Hox complex is made up of 39 genes distributed in paralogous clusters on four chromosomes (Hox A-D) [9]. In addition, it has been estimated there are >150 further 'non-clustered' homeobox containing genes [10-13]. Understanding the exact mechanism by which Hox genes exert their effects during development represents a key goal to deciphering the regulatory network underlying morphogenesis of the body plan.

One proposed mechanism for Hox gene action is that they act as 'selectors' whose homeodomain proteins subsequently activate a battery of 'effector' or 'realizator' genes that implement the effect on cellular identity [14]. Presumably, the existence of overlapping Hox expression domains would further promote the activation of a unique set of effectors. The complement of effectors that are activated or repressed may be further divided into those directly recognised by binding of the Hox protein (primary regulation) and those regulated by the effectors themselves (indirect or secondary regulation). The final readout of Hox activity on tissue patterning is likely to be a combination of both processes across a wide range of cellular machinery. Despite this being the prevailing view of Hox-driven cellular patterning, relatively few direct or indirect targets of Hox genes have been identified [15,16]. Although some studies have established direct Hox targets in specific systems, they are insufficient to account for the ability of Hox proteins to direct homeotic transformations [17-19].

A key feature of both *Drosophila *and vertebrate Hox clusters is spatial co-linearity where, although there are exceptions, the expression of each Hox gene along the AP axis of the embryo is reciprocal to their order along the chromosome [20,21]. The most striking parallel between vertebrate and *Drosophila *Hox gene function can be seen in the development of the hindbrain and spinal cord [15,22-26]. It is now well established that the hindbrain is patterned by the combinatorial action of the Hox transcription factor code. The developing vertebrate hindbrain is transiently patterned into a series of metameric units called rhombomeres (r) that are cell-lineage restricted compartments. This characteristic series of morphological segments prefigures the ordered establishment of several neuronal populations in the hindbrain, including motor, reticulospinal and second-order sensory neurons [24,25,27-29].

Within the hindbrain region of the neural plate (initially defined as *Gbx*2 positive), the Hox code is first set up under the influence of AP signals such as retinoic acid (RA) [23,30,31]. Given the distribution of Hox transcripts and their ability to act in a co-operative fashion, it has been suggested that individual rhombomere identity is conferred by a combinatorial code of the Hox proteins [25]. Functional evidence for the role of Hox genes in hindbrain patterning comes from interference studies on the *Hoxb1 *gene, which is normally highly expressed only in r4. Disruption of the *Hoxb1 *gene in mice leads to transformation of the r4 territory into an r2-like state [32], whereas retroviral-mediated over-expression of *Hoxb1 *in r2 causes homeotic transformation of r2 to r4-like in chick [33]. These and other examples suggest the loss of function of a Hox gene converts the territory it is normally expressed in to the identity usually associated with the remaining set of Hox genes [23,26,34-37]. The 'executive' function of Hox genes has also been described during neuronal specification along the AP axis of the spinal cord. Here, where Hox genes are also expressed at distinctive AP positions, they have been shown to regulate the establishment of the columnar and pool specification of motor neurons at both the brachial [38-40] and lumbar [41-43] levels [44].

Evidence is also emerging about some of the factors that act upstream of Hox genes to activate them at appropriate AP levels within the hindbrain. As well as an early influence of RA and cross-regulation between the Hox genes, upstream regulators include Mafb and Egr2. In addition to controlling segmentation of the neuroepithelium, these factors act in a parallel but related process to regulate the Hox genes. Thus, Mafb directly modulates expression of paralogue group 3 Hox genes in r5 [45,46], and Egr2 is a direct activator of both *Hoxa*2 and *Hoxb*2 and a repressor of *Hoxb1 *[47-50].

Whilst Hox proteins have been shown to determine the assignment of neuronal identity in the developing neural tube and some of the up- and downstream regulatory components are known, we have sought to expand the identity of the set of genes that show regionally restricted expression across the hindbrain. These differentially expressed genes may be part of the downstream effectors responsible for Hox patterning. We reasoned that the developing hindbrain represents the ideal model system to investigate the genetic controls underlying the emergence of fine distinctions in rhombomere identity and motor neuronal diversity, as manifested by such properties as selective growth cone navigation and cell body migration. For example, each rhombomere is morphologically distinct and thus accessible and there is a precise correspondence between the rhombomeres and domains of Hox gene expression. Accordingly, the set of genes activated in each rhombomere can be linked with one Hox combination (that is, r2 with *Hoxa2 *alone, r4 with *Hoxa2*, *Hoxb2 *and *Hoxb1*). As well as activating a set of downstream effectors, Hox genes are known to contribute to the maintenance of their expression domains via both auto- and cross-regulatory interaction. Furthermore, there are numerous reports of functional redundancy between members of the Hox gene family. Taken together, these factors can potentially complicate the search for the downstream effectors by genetic intervention and candidate screening approaches. For these reasons, we have chosen to generate a set of candidate genes from individual rhombomeres expressing their normal repertoire of Hox genes.

Using microarray and bioinformatic methodologies in conjunction with other confirmatory techniques, we report here a detailed and comprehensive set of potential Hox target genes in r2, r3, r4 and r5. These data are used to address several questions about the patterning of the developing hindbrain. First, what set of genes is significantly differentially expressed between r2–r5 of the developing hindbrain? Second, can these sets of genes be classified into genetic motifs that logically correspond to Hox-driven patterning? Third, can we derive insights from this dataset to enhance our understanding of the observed pattern of neurogenesis in the segmental hindbrain?

## Materials and methods

### Microarray experiments

**Figure 1:**
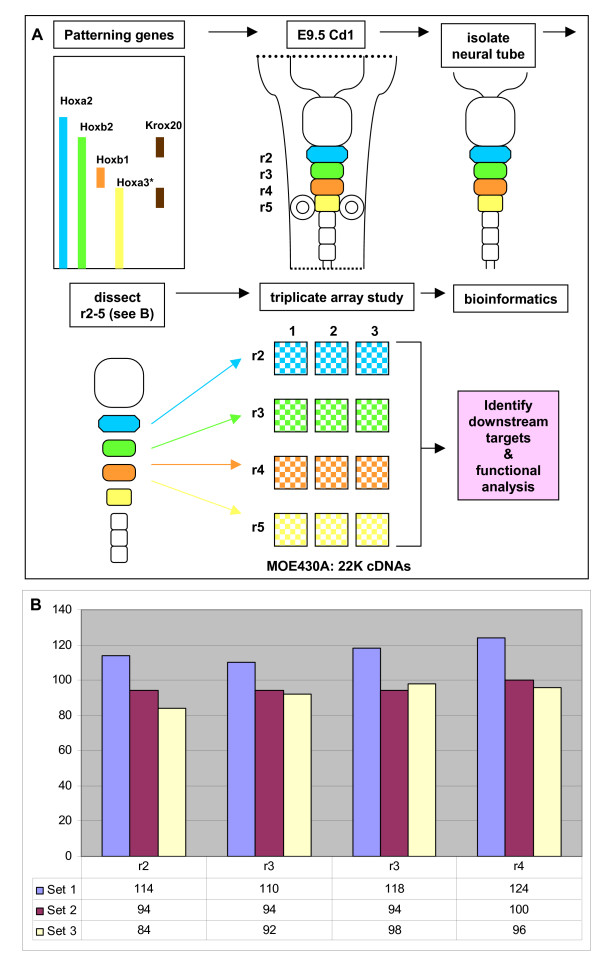
**Experimental strategy for the identification of genes differentially expressed across the embryonic day (E)9.5 hindbrain**. **(A) **Schematic diagram of the approach. Each rhombomere (r) is patterned by a unique combination of transcription factors whose expression domains are tightly correlated with rhombomere boundaries. Intact neural tubes were isolated from stage-matched CD1 litter mates by partial proteolytic digestion and subsequently dissected into individual rhombomeres. Each rhombomere was pooled with its equivalent and pools accumulated from individual litters and times were classified as sets 1–3 (i.e. biological triplicates). Individual sets from each rhombomere pool were processed and hybridised to Affymetrix MOE430A GeneChips. Genes whose expression differed significantly between rhombomeres, and thus candidates for Hox downstream targets, were identified by one way ANOVA. The expression of potential targets was interrogated by *in situ *hybridisation to E9.5 CD1 embryos. **(B) **The number of stage-matched rhombomeres combined is each set represented graphically.

Neural tubes from stage-matched embryonic day (E)9.5 CD1 mouse embryos were isolated from surrounding ectoderm and mesoderm as described in [51]. Individual rhombomeres (r2–5) were isolated by cutting precisely along the characteristic rhombomere boundaries (Figure 1A). To obtain sufficient total RNA for a microarray experiment, number-matched rhombomeres were collected in pools (Figure 1B). Total RNA was isolated from each pool as described in [51]. Tissue samples collected by the above criteria but on different occasions by the same operator were designated as biological replicates. Each rhombomere is represented by triplicate pools (denoted as sets 1–3) and the number in each set is given in Figure 1B. Labelled extracts were generated from 10 μg of total RNA by the Enzo (New York, NY, USA) T7-based 1 cycle protocol and hybridised to Affymetrix (Santa Clara, CA, USA) MOE430A GeneChips as per the manufacturer's instructions. The representation and efficacy of the labelled extract conversions was monitored by hybridization to Test 3 GeneChips prior to further use (data not shown).

### Bioinformatics

Probe level values were calculated from the raw data using the MAS5 algorithm embedded into the GCOS suite (version 1.2; Affymetrix). Data were analysed using the GeneSpring package (version 7.3.1; Agilent Technologies, Wokingham, Berkshire, UK). The suitability of the expression data sets for inclusion in the analysis and the overall relationship between and within the biological replicates was assessed using quantile plots, principle components analysis (PCA) and hierarchical clustering, respectively (data not shown and Additional file 1A,B). Differential expression between samples was determined by the application of a multi-step process. Samples were first normalised to the 50th percentile (median) across the entire expression dataset and then each gene was normalised to the median of its own expression across each rhombomere. Thus, gene expression profiles are scaled and centred about 1, where 1 represents the median of a gene's expression across the experiment. In this way, a gene expression value greater than 1 is classified as enriched whereas a value of less than one is depleted (or absent) in a particular rhombomere with respect to the median level of that gene's expression across the whole experiment. Prior to statistical analysis, genes classed as being not expressed (that is, absent in three of three biological replicates) or not varying their expression above a twofold level in any of the rhombomere samples after averaging their expression in the same rhombomere were removed from the analysis (defined here as 'non-changing' genes). Genes with low raw values across all of the rhombomeres (between 0.01 and 20) were also removed prior to statistical analysis. Using the remaining set of genes (defined here as 'changing and reliable' genes), genes whose expression levels differ significantly between each rhombomere were determined by one-way analysis of variance (ANOVA; *p *= 0.05). Statistically different genes were functionally classified using a combination of Gene Ontology (GO) criteria and other molecular descriptions derived from UniGene, GenBank and Entrez Gene databases. Genes were clustered into potentially co-regulated groups using both unsupervised and supervised approaches including self-organising maps.

### Molecular analyses

Whole mount *in situ *hybridisation with digoxigenin-labelled riboprobes was performed as described in [51]. Embryos analysed by sectioning were embedded in 20% gelatin and fixed in 4% paraformaldehyde/phosphate-buffered saline for at least 3 days. Sections were cut at 40 μm on a vibratome and mounted in 80% glycerol/phosphate-buffered saline before being photographed using a digital camera. Reverse transcription PCR was performed as described [51].

### Plasmids

Plasmids were obtained from the ImaGene resource centre (Berlin, Germany) by matching their unique Affymetrix identifier to the corresponding sequence-verified cDNA.

## Results

### Experimental strategy

As a prelude to establishing Hox regulatory networks, we sought to determine the complete transcriptional repertoire of individual rhombomeres in the E9.5 mouse hindbrain by microarray analysis (Figure 1A). At this developmental stage the hindbrain affords a unique opportunity to monitor the effects of specific Hox patterning cues on each rhombomere and the neuronal populations that are specified within them. We chose to examine the battery of genes expressed across the hindbrain at E9.5 as it is possible that genes that act both upstream (for example, *Egr2*) and downstream (for example, *EphA4*) of Hox genes will be expressed at this time, thus maximising our chances of gaining insights into the global mechanism of Hox action.

These studies have been performed in wild-type animals so that the derived expression profiles could be correlated with the endogenous Hox patterning cues and levels. This is in contrast to studies that have sought to identify Hox responsive genes by adopting either genetic loss- or gain-of-function approaches [17,18,52] and examining the subsequent transcriptional profile of the 'mutant' territory. Whilst these approaches present certain advantages, there are limitations in the interpretation of the output due to several factors, such as tissue re-specification and penetrance of the mutant phenotype. For example, in the hindbrain Hox proteins are known to cooperate in extensive cross-regulation, and loss of one Hox protein can lead to the ectopic activation of another that obscures the potential to identify the change in the downstream target profile of the intended Hox gene. Furthermore, over-expression strategies may be complicated by non-representative levels of the Hox protein or inappropriate availability of the correct repertoire of Hox co-factors required to mimic Hox activity at an ectopic site. Also in this case, rhombomeres signal to each other in a bi-directional planar fashion that will likely affect the transcriptional repertoire of both source and sink fields (for example, Eph-Ephrin interactions [53]). Therefore, using hindbrains where the Hox code has been generically perturbed may result in a more widespread change in the transcriptional profile of the hindbrain segments than anticipated, making it more difficult to begin to define the 'normal' genetic hierarchies.

Thus, we provide data where the global gene expression in a normal tissue (that is, neuroepithelium of the segmented hindbrain) can be tightly attributed to its endogenous patterning influences (the combinatorial Hox code). The set of genes uniquely associated with each segment can thus be derived from parallel comparisons of the gene expression profiles of each rhombomere. The set of genes employed to direct general 'rhombomeric' identity will be common to each gene expression set and is not dealt with here.

### RNA populations can be isolated exclusively from individual rhombomeres

To examine the profile of genes expressed exclusively by r2–5, neuroepithelial tissue was explanted in the absence of adherent tissues (for example, neural crest, epidermal ectoderm, or mesoderm). The identity of each dissected rhombomere pool was confirmed by RT-PCR for the presence or absence of known markers (for example, *Hoxb1 *expression in r4 and *Egr2 *in r5; data not shown). Having established that the dissected tissues were representative of the source material, we next analysed the global gene expression set of each rhombomere using microarrays that constituted 22,690 previously characterised mouse cDNAs (Affymetrix MOE430A GeneChip). To avoid any potential biases associated with extensive amplification methodologies (for example, [54]) we collected sufficient neural tissue to generate 10 μg of total RNA to perform a standard T7-based labelling procedure. Accordingly, each set consisted of hundreds of stage-matched rhombomeres (Figure 1B). Using standard approaches, we generated expression data from triplicate sets (sets 1–3) of independently collected rhombomere pools that complied with the established quality control metrics for representative Affymetrix array experiments (that is, background, raw Q and noise levels).

### Biological replicates show consistent transcriptional trends

First, the distribution of gene expression values across the whole chip was monitored before and after normalisation by quantile grouping (box plots) and shown to be similar for each set of biological replicates (data not shown). We next examined the relationship between the biological replicates using PCA. Using the set of genes defined as 'changing and reliable' to examine the underlying trend, PCA demonstrated that biological replicates were markedly more similar to each other than the replicates from a different rhombomere sample (Additional file 1A). A similar observation was seen using hierarchical clustering on the same gene set (Additional file 1B). Together, these findings indicate the reproducibility of the biological replicate datasets and are indicative of underlying transcriptional differences between the rhombomeres.

### Expression profiles are characteristic of neural tissue

Prior to statistical data analysis, we first examined the dataset for the presence of known neural marker genes. In the context of gene expression, perhaps the best characterised aspect of neurogenesis in the hindbrain is the specification of the cranial motor neurons [24,55]. Accordingly, the expression of genes associated with the establishment of motor neuron progenitor domains (that is, *Nkx2.2*, *Nkx2.9*, *Gli1-3*, *Pax6*) were recorded at high levels throughout all the samples (data not shown). Later determinants of cranial motor neurons (that is, *Isl1 *and *Phox2b*) were also observed.

### Proof of principle: Hox genes and known downstream targets are identified

Of the 22,690 probe sets described on MOE430A, 13,820 genes were assigned as being present as defined by Affymetrix criteria in at least 3 of the 12 samples. Of these, 1,381 genes were found to vary their expression levels between the rhombomeres and have a raw value >20. The genes changing their expression levels most significantly between r2–5 (381 in total) were determined by ANOVA with a *p*-value cutoff of 0.05 (Additional files 2A and 3). Thus, this is the set of genes that are most likely to show detectable variations in their expression levels and constitute genes subject to patterning influences. Functional classification of the annotated gene list, which may represent the Hox effectors or other co-regulated genes, was performed by comparison to the GO molecular function database and is summarised in Additional file 2B,C. The efficacy of the screen described here was confirmed by the presence of known differentially expressed genes with the pool of statistically significant identified genes.

**Figure 2:**
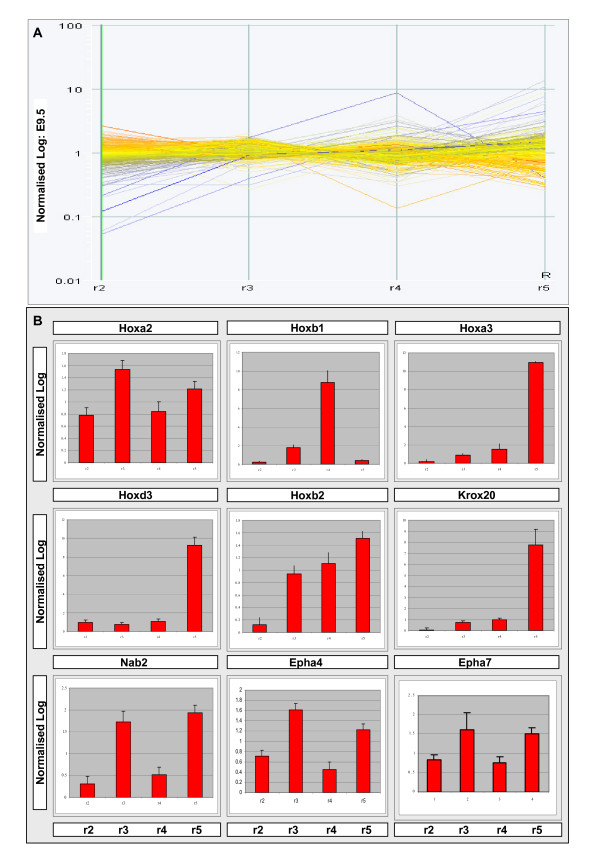
**The set of candidate effectors or upstream activators of Hox-mediated patterning**. **(A) **A summary of expression profiles (r2–r5) of the genes identified by ANOVA (*p *= 0.05). Each line represents the expression of one gene where 1 is equivalent to the median of each gene's expression across the experiment. Therefore, >1 predicts enriched expression whereas <1 suggests reduced expression relative to the median. Normalised values for each gene represented are detailed in Additional file 3. **(B) **GeneChip expression profiles of the hindbrain patterning genes are concordant with the endogenous expression domains. Subtleties in the expression values can also be detected. For example, *Hoxa2 *expression is elevated in r3 and r5 and this is consistent with its known transcriptional activation in this region by Egr2. Together, these data show that the methods used here generate a faithful depiction of known gene expression across the hindbrain. At E9.5, *Egr2*, the primary determinant of r3 and r5 territories, is expressed only in r5 (Figure 4A'-A"'). The expression of some of the known Hox/Egr2 downstream targets (for example, *Nab1*/*2*) is accurately represented. Data represented as mean normalised value  +/- SEM.

Firstly, the expression of the Hox genes themselves was faithfully mirrored in the gene expression profiles generated from the processed data (Figure 2B). As is observed by *in situ *hybridisation [24], *Hoxa2 *was represented as being expressed throughout the hindbrain whereas *Hoxb1*, *Hoxb2*, *Hoxa3*, and *Hoxd3 *were restricted to their appropriate domains. *Egr2*, the key determinant of r3 and r5 identity, was also accurately reflected in the array profiles as well as some of its known molecular effectors (*Nab1*, *Nab2*, *Epha4 *and *Epha7 *[56,57]). The data derived here are, therefore, a precise readout of both even (r2 and r4) and odd (r3 and r5) rhombomere identity. Furthermore, subtleties in the expression levels of the Hox genes across the hindbrain were also recorded. *Hoxa2 *is expressed throughout r2–5 but its expression in r3 and r5 is enhanced by an Egr2-dependent regulatory mechanism [48,50]. This differential enhancer usage between odd and even compartments is revealed here by the enriched array signal recorded for *Hoxa2 *in r3 and r5 when compared to its expression in r2 and r4 (Figure 2B, *Hoxa2 *panel). These observations lend strong support to the array data presented here being reflective of endogenous positional changes in gene expression along the AP axis of the hindbrain.

Further investigation of the differentially expressed gene list revealed that patterns of gene expression associated with discrete cell populations within rhombomeres were also faithfully represented. In addition to its unique Hox code, each rhombomere has a characteristic population of cranial motor nuclei associated with it [23-25,55]. For example, the motor nuclei of the trigeminal (V) nerve are derived initially from r2 and r3, whereas the facial motor nuclei develop in r4 and r5 [24,55,58]. Furthermore, the timing of neurogenesis of the cranial motor nuclei within the rhombomeres is not equal [58]. At E9.5, the differentiation of V and VII motor nuclei in r2 and r4, respectively, is underway whereas in r3 and 5 it has yet to commence. Accordingly, differences in gene expression can be associated either with cues that impart molecular identity or with those that are associated with the regulation of neuronal differentiation.

Other confirmed gene expression changes include *Gata2 *and *Gata3*, both determinants of r4 motor neuron identity [59] that are found exclusively in the list of r4 enriched genes, and *Nab1 *and *Nab2*, downstream targets of Egr2 [56] that are in r3 and r5 gene lists. Several other genes presented here have also been described previously to be expressed in regionally restricted patterns across the developing hindbrain (for example, *RARα*, *Ebf1 *[24], and *bHLHb2 *[60]).

### Verification of candidate genes enriched in even rhombomeres

Following validation of the dataset, we next sought to identify genes whose transcription was enriched in r2, r4 or both. The observed differences in cell mixing and neuronal architecture (that is, motor neuron composition, interneuron periodicity and presence of exit points) suggested that there are multiple genetic cues specific to, or differentially regulated in, these territories [23-25,55]. Candidate genes were selected based upon their reported expression profiles across the hindbrain (for example, enriched in r2 compared to r4 or enriched in r2 and 4 compared to r3 and 5) as well as their predicted function and integration into known signalling events within r2 and r4. A fold change filter was not applied as a criterion for difference in levels of gene expression (for example, >2-fold between rhombomeres); therefore, as long as a difference in gene expression between two rhombomeres was statistically significant, it was included in the set of genes described here. We chose to validate the expression of these genes by *in situ *hybridisation to define the set of expressing cells both across the hindbrain and within a rhombomere.

**Figure 3:**
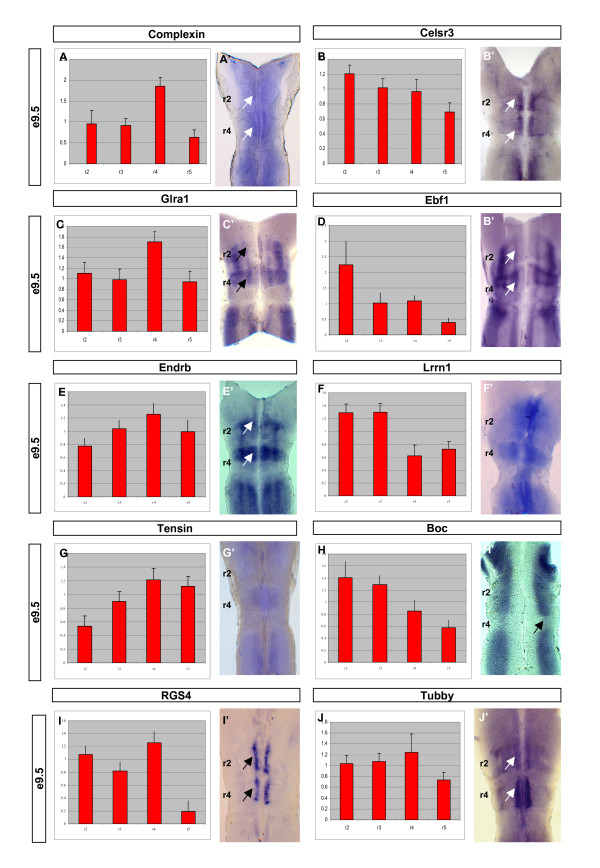
**Genes enriched in even-numbered rhombomeres (r2 and r4)**. **(A-J) **The normalised expression value is represented graphically for each rhombomere +/- SEM. **(A'-J') **Hindbrain flat mount preparations of *in situ *hybridisations of the corresponding gene. Arrows mark the enriched sites of expression in the r2 and r4 territories. The genes were selected for verification based upon their molecular functions and predicted expression profiles. Generally, the expression profile is consistent with that predicted by the microarray data.

The expression of *Complexin*, *Slc12a5*, *Glra1*, *Endrb*, *Tensin*, *RGS4*, *Celsr3*, *Ebf1*, *Boc *(*Biregional cell adhesion molecule*), and *Tubby *was examined in the E9.5 hindbrain (Figure 3A'–J'). We found that each gene was expressed in a variety of patterns that were enriched in the even-numbered rhombomeres. Closer inspection revealed that each candidate was expressed in a pattern closely correlated with that predicted from the array profile (Figure 3A–J, represented as histograms ± 1 standard deviation). However, there were genes where the array profile was not concordant with the observed *in situ *pattern (for example, *Celsr3 *(Figure 3B) and *Tubby *(Figure 3J)). This suggests a nonlinear response of the array output to the amount of mRNA originally derived from the rhombomere. Genes that are expressed only weakly within a rhombomere (for example, *Tensin *(Figure 3G)) or expressed in only a subset of cells within a rhombomere (for example, *Celsr3 *(Figure 3B) and *Tubby *(Figure 3J)) may be particularly susceptible to discrepancies between array output and actual mRNA levels. However, it is also possible that the probe sets on the microarray for these particular genes are relatively poor at binding their cognate labelled mRNAs in a linear manner. Consequently, the output of the array screen serves as a guide to expression levels rather than an absolute read out. Some of the candidates were enriched throughout either r2 or r4 (that is, *Slc12a5 *and *Tensin*) whereas others were restricted to particular cell populations with these segments (*Celsr3*, *RGS4*, *Complexin*, *Tubby*). In most cases, expression was seen in discrete locations outside the r2 and r4 territories. Reciprocally, we looked for genes that were down-regulated in even-numbered rhombomeres compared to the rest of the hindbrain and, therefore, were candidates for repression by Hox activity. Here, Boc (Figure 3H', black arrow) and Lix (data not shown) were seen to be exclusively reduced in r4. The prevalence of genes whose expression was not exclusively restricted to individual or pairs of rhombomeres suggests gene expression across the hindbrain may be a combination of discrete and enriched transcriptional events.

### Verification of candidate genes enriched in odd-numbered rhombomeres

Egr2 is a zinc finger transcription factor expressed in r3 and r5 that is a key determinant of these territories [47,61-63]. As well as demarcating these regions, Egr2 is also know to directly regulate the r3 and r5 expression of EphA4, a receptor tyrosine kinase that is responsible, at least in part, for preventing cell mixing with adjacent rhombomeres [57,64]. Egr2 is also known to regulate the expression of Hoxa2 and Hoxb2 in these rhombomeres [48,50]. Furthermore, a basic leucine zipper transcription factor (Mafb/Kriesler) that is expressed in r5 and r6 is also known to play a role in establishing r5 identity [45,65-68]. The synergistic interaction of these factors imparts the identities of r3 and r5. However, despite detailed knowledge of how these components cross-regulate [69], very little is known about the set of downstream genes that they co-ordinately regulate.

**Figure 4:**
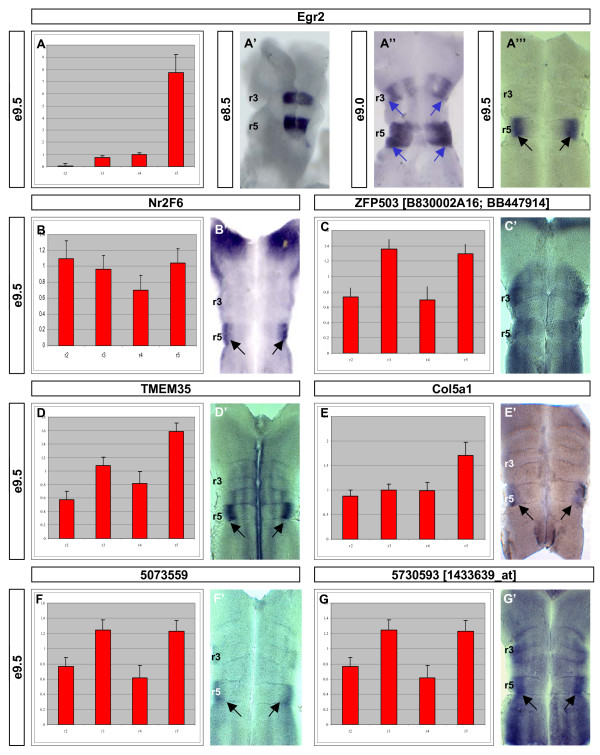
**Genes enriched in the odd-numbered rhombomeres (r3 and r5)**. **(A-G) **The normalised expression is represented graphically for each rhombomere +/- SEM. **(A'-G') **Hindbrain flat-mount preparations of the corresponding gene. **(A'-A"') **The r3/5 patterning gene has a dynamic expression pattern in the hindbrain between E8.5 and E9.5 (blue arrows). At the time of the tissue harvesting (E9.5), *Egr2 *was down-regulated in r3 and restricted to dorsal r5 territories (A"', black arrows). Genes potentially enriched in odd-numbered rhombomeres were selected for verification based upon their molecular functions and predicted expression profiles. The expression profiles are consistent with the derived microarray output.

Using the validated dataset described here, we searched for other genes whose expression was selectively regulated in the r3 and/or r5 region. Egr2 shows a dynamic expression in r3 and r5 as development proceeds [61]. To potentially correlate the expression of candidate genes with known patterning cues, we first described the expression of *Egr2 *between E8.5 and E9.5 (Figure 4A'–A'"). Whilst Egr2 is expressed throughout r3 and r5 at E9 (Figure 4A", blue arrows), by E9.5 its expression is restricted to a dorsal subregion of r5 (Figure 4A"', black arrows). Consequently, using the same functional criteria described above, we searched for genes whose expression was enriched in both r3 and r5 (that is, related to the first wave of Egr2 activity) and in r5 alone (that is, possibly related to later Egr2 activity) by *in situ *hybridisation.

Parallel to the case seen for r2 and r4, expression of the candidate genes included both enrichment in the odd-numbered rhombomeres (*ZFP503*, *TMEM35*, 5073559 and 5730593) and segmentally restricted expression (*Nr2F6 *and *Col5a1*) (Figure 4B–G). However, the majority of the genes examined here showed an enriched region of expression in the locality of the E9.5 domain of Egr2 expression (Figure 4B',D'–G', black arrows). These findings may suggest that Egr2 both activates specific gene expression and modulates the levels of more generically expressed genes (see Discussion). Again, the molecular function of the genes enriched in the odd-numbered rhombomeres spans a wide range and suggests the involvement of Egr2 in multiple mechanisms (see below).

### Functional classification of candidate Hox downstream genes

The functional readout of Hox genes can be gauged by monitoring the diversity of molecular functions, cellular locations and biological processes that are represented by the effectors. We chose to assimilate the functions of the effectors using the GO criteria based upon experimentally established functions reported in various databases.

Of the 255 genes annotated for GO biological processes, most were classified as being involved with cellular (243; for example, cell communication and differentiation) or physiological (206; cell metabolism and death) processes (Additional files 2B and 3). The GO is a redundant classification where one gene may be listed under several groupings. Second most abundant were those genes involved with regulation of biological processes (87; regulation of growth and enzyme activity) and development (84 morphogenesis and pattern specification).

To help identify candidate genes of interest (for example, those encoding cell adhesion molecules) the potential effectors were also grouped according to their cellular location (Additional files 2C and 3). Of the 250 GO annotated genes, 227 were registered as being cellular, 140 organelle associated, 72 in the extracellular region, 51 in protein-protein complexes and 19 as components of the extracellular matrix. Proof of principle was indicated by the assignment of the Eph receptors to the cell membrane and extracellular space. A potentially significant observation to arise from this analysis, currently under investigation, was the presence of protocadherin 18 (r4-enriched) and cadherin 2 (r5-enriched) in the dataset, since these are known to be important regulators of cell adhesion and recognition in the developing nervous system.

Thirdly, we organized the genes by their GO molecular function (Additional file 3). This particular sorting is indicative of the types of proteins encoded by the set of differentially expressed genes. The largest category by number comprised those genes listed as binding-related (209; for example, protein-, microfibril-, phosphate-, hormone-binding) and catalytic activity (88; for example, oxidoreductase activity). Forty-six genes were defined as having transcriptional regulator activity and a further 40 as being involved in signal transduction. We assimilated the information derived from each of these classifications and used it to help select our set of candidate genes to pursue further (see below).

### Identifying co-regulated genes: r4

**Figure 5:**
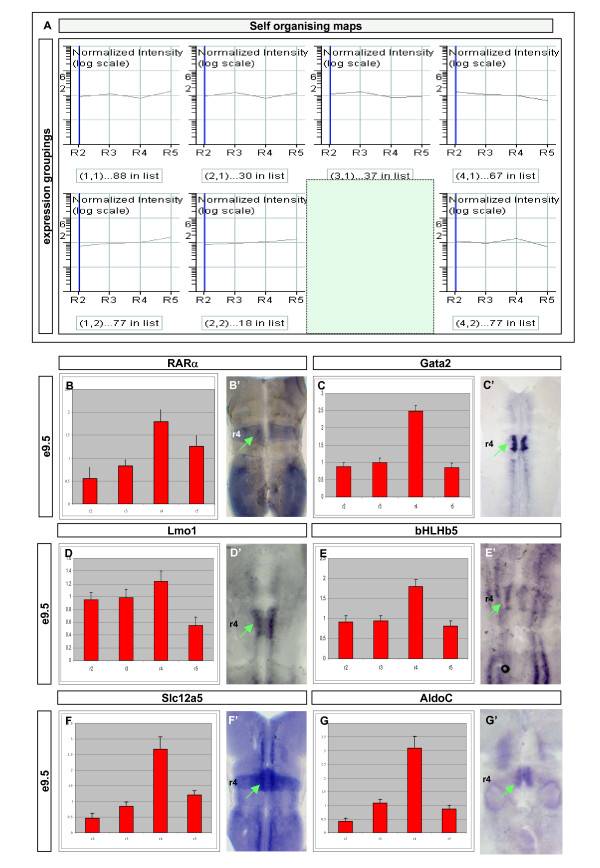
**Self-organising maps (SOMs) cluster the set of differentially expressed genes into related profiles and predict likely downstream targets in each rhombomere: r4 enriched**. **(A) **To uncover underlying trends in the global expression profiles, genes were clustered using SOMs. The 'average' profile of the gene expression pattern contributing to a particular group is shown in each cluster. The number of genes following this pattern is given under each graph. The cluster highlighted in green (that is, enriched in r4) was selected for further study. **(B-E') **Verification of expression patterns of a selection of genes identified in the r4-enriched SOM. Adjacent to each graph showing a relatively high r4 expression value is a hindbrain flat mount preparation of an *in situ *hybridisation of the corresponding gene in an E9.5 embryo. In each case, the expression of the gene was either up-regulated or significantly enriched in either all or part of r4. Relative expression and *in situ *patterns are shown for *RARα*, *Gata2*, *Lmo1*, *bHLHb5*, *Slc12a5*, and *AldoC*. Each of these genes was derived from the same SOM cluster. *Gata2 *is a known direct downstream target of the r4 patterning gene *Hoxb1*. The normalised expression is represented graphically for each rhombomere +/- SEM.

**Figure 6:**
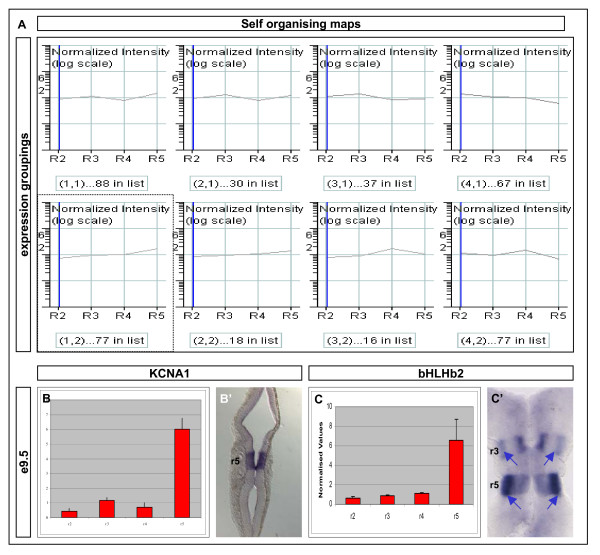
**Self-organising maps cluster the set of differentially expressed genes into related profiles and predict likely downstream targets in each rhombomere: r5 enriched**. **(A) **To uncover underlying trends in the global expression profiles, genes were clustered using SOMs. The 'average' profile of the gene expression pattern contributing to a particular group is shown in each cluster. The number of genes following this pattern is given under each graph. The cluster highlighted in yellow (that is, enriched in r5) was selected for further study. **(B-C') **Verification of expression pattern of a selection of genes identified in the r5-enriched SOM. Adjacent to each graph showing a relatively high r5 expression value is a hindbrain flat mount preparation of an *in situ *hybridisation of the corresponding gene in an E9.5 CD1 embryo. In each case, the expression of the gene was either up-regulated or significantly enriched in either all of or part of r5. Relative expression and *in situ *patterns are shown for *Kxna1 *and *bHLHb2*. Each of these genes was derived from the same SOM cluster. The normalised expression is represented graphically for each rhombomere +/- SEM.

**Figure 7:**
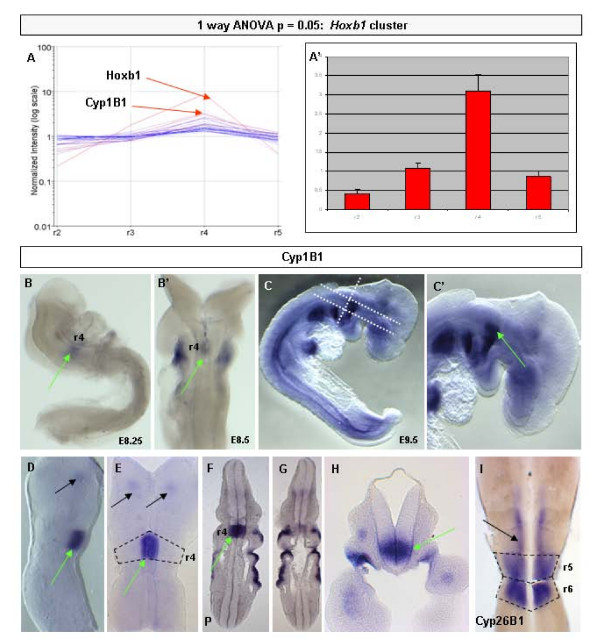
**(A,A') Analysis of the candidates from the rhombomere 4 (r4)-enriched self-organising map predicts *Cyp1B1 *to have r4-restricted expression**. **(C-H) ***In situ *hybridisation reveals that *Cyp1B1 *is expressed in the ventral region of r4 at embryonic day (E)9.5 (green arrows). White dashed lines (C) represent plane of section in F, G & H. Black dashed line represent r4 boundaries (E) or r4 7 5 boundaries (I) **(B,B') **Analysis of embryos at E8.5 show that Cyp1B1 is switched on shortly after the induction of *Hoxb1 *in r4 (green arrows). Functional studies of *Cyp1B1 *suggest that it drives a RALDH2-independent retinoic acid signalling pathway [70]. **(I) **Members of the *Cyp26 *gene family (for example, *Cyp26B1*; black arrow) modulate RA-signalling in the hindbrain to determine appropriate Hox expression (for example, see [71]). The normalised expression is represented graphically for each rhombomere +/- SEM.

To identify potential regulatory networks, we next asked what groups of genes could be isolated by using either supervised or unsupervised clustering techniques. In particular, we focussed on genes whose expression followed a similar profile to *Hoxb1*, the key determinant of r4 identity. Using either quality threshold clustering (data not shown) or self-organising maps (Figures 5A, 6A and 7A), we parsed the gene list into groups that showed similar expression profiles. The underlying assumption is that genes with similar expression profiles may be operating in the same regulatory network. Analysis of the profile up-regulated in r4 revealed a set of genes containing, amongst others, *Hoxb1 *and *Gata2*. We chose to examine the expression of two other transcriptional regulators in this group, *Lmo1 *and *bHLHb5*. In each case, *in situ *hybridisation detected strong expression in r4 at E9.5, as predicted by the profile (Figure 5D',E', green arrow). In both cases, transcripts were not distributed evenly throughout r4 but instead appeared to be restricted basally, to the region of motor neuron specification. *bHLHb5 *was also seen to be expressed in longitudinal stripes through the alar hindbrain (Figure 5E'). These data promote the view that Lmo1 and bHLHb5 play a role in early patterning of the branchiomotor neurons born in r4 (VII and VIII). Their involvement in the established hierarchy of motor neuron specification is currently under investigation. Other genes in the r4 list, such as *Gpx3 *(data not shown), *AldoC*, *Slc12a5 *(Figure 5F',G', green arrows), and *Glra1 *(Figure 3C), were also validated, although their function in the patterning of r4 remains unclear.

Further detailed analysis of the identity and function of other candidate (as defined by the GO database) revealed that a member of the cytochrome p450 family (*Cyp1B1*) showed an expression profile related to *Hoxb1*. This observation was of interest given that other members of the cytochrome p450 family (that is, the Cyp26s) have a well documented role in establishing correct hindbrain patterning via their role in attenuating RA signalling [30] whereas we have recently shown that Cyp1B1 is capable of synthesizing RA [70]. With these insights, we chose to examine *Cyp1B1 *expression in detail during mouse hindbrain development.

Firstly, we examined the expression of Cyp1B1 at E9.5 to see if it corroborated the array profile (that is, is exclusively expressed in r4). At this time, *Cyp1B1 *transcripts were found to be expressed in a ventral domain throughout r4 (Figure 7C,C', green arrow) [71]. As predicted, *Cyp1B1 *was not detected in r2, r3 or r5. However, *Cyp1B1 *transcripts were seen in a small patch of cells in ventral r1 (Figure 7D, E, black arrows). Longitudinal sections through the E9.5 hindbrain confirmed that *Cyp1B1 *was restricted to r4 as defined by the characteristic morphological boundaries (Figure 7F, green arrow). We next looked at E8–8.5 embryos to assess if *Cyp1B1 *was detectable from the onset of *Hoxb1 *in this region. Here, r4-specific expression of *Cyp1B1 *was seen prior to neural tube closure (Figure 7A,B, green arrow). Thus, the onset and differential AP expression of *Cyp1B1 *is consistent with it being activated by Hoxb1 in this region, although the dorsoventral restriction of *Cyp1B1 *in r4 (Figure 7H, green arrow) is suggestive of another regulatory mechanism (for example, repressive dorsal-originating signals). Furthermore, as development proceeds, the expression of *Cyp1B1 *remains localised to r4 but is progressively refined to the r4 motor neuron progenitor domain (data not shown).

We propose that Cyp1B1 may be involved in RA signalling in the basal plate of r4 during establishment of early hindbrain pattern. The observation that *Cyp1B1 *is expressed in ventral r4 is also consistent with the observation that there is a region of Raldh2-independent RA signalling present in a coincident ventral domain of caudal mouse hindbrain at this time [72]. The localised expression of *RARα *in r4 (Figure 5B) lends further support for an important role of RA during the patterning of this territory. Thus, we have identified a potentially important signalling pathway during hindbrain development and this may begin to give an insight into how the Hox regulatory network may be translated into differences in cellular identity and behaviour.

### Identifying co-regulated genes: r5

To identify key effectors of r3 or r5, we sought to distinguish the group of genes co-expressed with *Egr2*, using identical approaches to those above (Figure 6A). There were 77 genes seen to have a similar array pattern to *Egr2 *(normalised values: r2 = 0.535, r3 = 1.129, r4 = 1.142, r5 = 15.93), which at E9.5 is no longer expressed in r3 (Figure 4A"'). Other validated genes in this group were *Hoxa*/*b*/*c3*, *Nab1 *and *Nab2 *(data not shown). To see if any other transcription factors operated downstream of Egr2, we restricted our analysis to these members of the group. In particular, *Stra13 *(*DEC1*/*bHLHb2*) showed an array profile suggestive of expression in r3 and r5. *In situ *hybridisation showed transcriptional activation throughout r3 and r5 at E9.5. However, consistent with *Egr2 *expression, dorsoventral variations in the intensity of *Stra13 *expression were noted (Figure 6C', blue arrows). Some of the other candidates identified as being co-regulated with *Egr2 *were suggestive of the acquisition of later aspects of neuronal identity. For example, potassium voltage-gated channel, shaker-related subfamily, member 1 was found to have expression restricted to r5 of the E9.5 hindbrain (Figure 6B').

### Transcriptional relationships between rhombomeres

**Figure 8:**
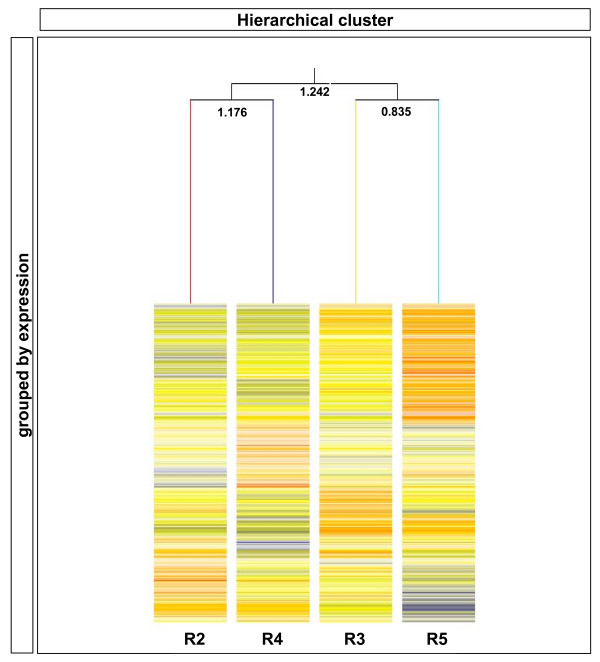
**Hierarchal clustering of the dataset reveals the level of genetic relatedness between rhombomeres**. Data sourced from the validated dataset shows that rhombomere 2 (r2; *Hoxa2*) and r4 (*Hoxa2*, *Hoxb2*, *Hoxb1*) are more related to each other than the r3 (*Hoxa2*, *Egr2*) and r5 (*Hoxa2*, *Egr2*, *MafB*) territories. These findings are consistent with previously described expression patterns and other data examining the cellular behaviour of these territories. Each gene in the cluster is represented by a single box at the same level and relative expression values in each rhombomere are depicted by the actual colour. Blue = underrepresented; yellow = equivalent to; and red = enriched with respect to the median expression value of that gene in all samples. The confidence scores are reported adjacent to each branch.

Observations of neuronal architecture in conjunction with cell labelling/mixing techniques and gene expression data support the notion that r2 is most similar to r4 and that r3 is most similar to r5. Using the validated dataset derived here, we can obtain a precise measure of the genetic relationships between individual rhombomeres and see if these assertions hold true across widespread transcriptional patterning. To this end, we chose to assess the relatedness of rhombomeres across the total set of genes that show a variation in expression across the hindbrain. Hierarchical clustering on the 'changing and reliable' dataset confirmed the widely held view that r2 is molecularly more similar to r4 (distance 1.176) and r3 more similar to r5 (distance 0.835) than the pairs (r2 and r4 compared to r3 and r5) are to each other (distance 1.242) (Figure 8).

**Figure 9:**
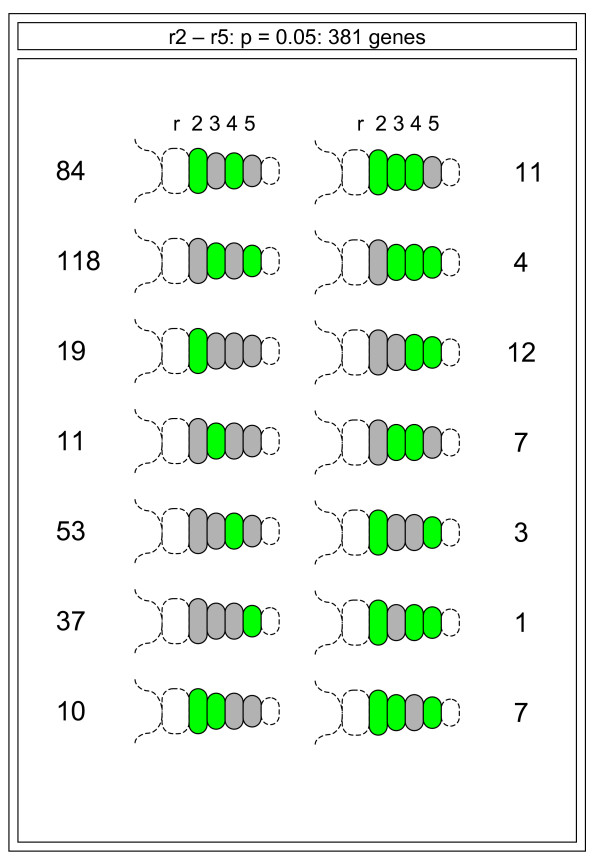
**Schematic diagram of an embryonic day 9.5 hindbrain showing all the variations in gene expression motifs**. Green represents any rhombomere (r) where the expression of a gene is greater than its median across r2–r5 of the hindbrain. All possible combinations of gene expression and segmental motifs were seen, suggesting that hindbrain patterning is the summation of numerous segmentally regulated events. Variations in the numbers observed for each motif suggest the dominant mechanisms that drive hindbrain patterning.

Additionally, using the information from differentially expressed gene lists, we have classified the set into 'segmental motifs' (Figure 9) based upon which rhombomere the gene is enriched in. Many of the groupings observed are readily understandable in the context of Hox gene activity. For example, many of the genes have expression that is enriched (either up-regulated or exclusively expressed) in r4 (55 genes; Figure 9). Similarly, expression groupings associated with r3 and r5 can also be ascribed to the patterning influences of Egr2 or related networks. Overall, this validated dataset and its division into discrete expression categories provides a valuable resource from which we can begin to explore the functional output of the combinatorial Hox code or other patterning influences acting on the early hindbrain.

## Discussion

Hox genes encode evolutionarily conserved homeodomain-containing transcription factors that have profound importance in the acquisition of regional and cell identity during development. However, the set of instructions encoded by a single or a combination of Hox proteins remains incomplete. We have established that the compartments associated with the developing hindbrain, whose identity is governed by the Hox code, show considerable differences in their transcriptional profiles. The set of genes defined as being differentially expressed across the hindbrain provide a large database of potential Hox effectors. These findings and their implications are discussed below.

### The search for Hox effectors

Several approaches employed across evolutionarily diverse animal models have been adopted in the search for Hox effectors. These methodologies can be broadly classified into those monitoring the consequences of genetic gain- or loss-of-function of Hox activity, mutation screens, predictive promoter networks, location analysis studies, and differential/subtractive screens [15]. Whilst each process has associated advantages and flaws, we have chosen to survey the expression of individual rhombomeres in the wild-type E9.5 hindbrain and to discern the potential cohort of Hox effectors by comparative analysis of the microarray-based data generated. This strategy has several advantages over previously described methods. First, expression of the genes identified can be directly correlated with normal levels of Hox activity rather than the potential complications associated with the penetrance of gain- or loss-of-function techniques. Secondly, our approach allows for the systematic survey of multiple tissues under the regulation of Hox activity (r2–r5) and the capacity to assess how different they are from one another simultaneously. Thirdly, we can determine the transcriptional profiles of regions patterned by one (for example, r2, *Hoxa2*) or multiple (for example, r4, *Hoxa2*, *Hoxb1*, *Hoxb2*) Hox inputs. Together, these factors present an attractive system to begin to define the repertoire of Hox effectors. However, given that Hox gene activation occurs around E8 and that the screen was performed at E9.5, there is the potential that multiple intermediate steps of regulation have occurred to pattern the hindbrain. Thus, our observed readout may be a combination of direct or indirect Hox targets, genes expressed as the result of other patterning influences (for example, RA) and co-regulated genes in the hindbrain.

### Proof of concept: known patterning genes are identified

The validity of our approach was confirmed by the identification and correct rhombomeric assignment of key known patterning genes in the E9.5 hindbrain. Data derived from the microarray study showed that each Hox gene known to be expressed in the hindbrain at E9.5 (*Hoxa2*, *Hoxb1*, *Hoxb2*, *Hoxb3*, *Hoxd3*) was appropriately classified, thus confirming both the precision of the initial rhombomere dissections (Figure 1A) and the subsequent readout of the microarray study. Similarly, the key upstream determinant of r3 and r5 identity, *Egr2*, was also assigned to the correct regions (Figure 4A).

Closer analysis of the list of differentially expressed genes reveals that certain genes known to be regionally restricted in the developing hindbrain are missing. For example, *Mafb*, which is expressed in the r5/6 territory and is a key determinant of its identity [66], does not appear in the list of differentially expressed genes. Two independent probe sets for this gene are present on the array but each failed to register statistically different gene expression values across the hindbrain. Although the reasons for this are unclear, it suggests that the experiment described here is susceptible to false negatives. Another example of a notable omission is *Cyp26b1*, a key modulator of RA in the caudal hindbrain, which is highly enriched in r5 and r6 but is not present in the set of genes generated here. However, inspection of the probe set on the MOE430A array shows that although well-characterised, *Cyp26b1*-specific sequences are not present. Thus, whilst the data described here provide a comprehensive list of the potential Hox effectors, the total pool is likely to be larger still. To this end, we have also duplicated this experiment using the MOE430B chip and identified several hundred further uncharacterised differentially expressed genes (data not shown). Compilation of these two datasets with subsequent analysis will be able to give a true indication of the proportion of known regionally restricted genes that were recovered by the screen.

### Known regulatory networks are predicted by clustering processes

Expression profiles that are similar in nature to one another are often used as predictors of co-ordinate gene regulation and the candidates are thus inferred to be in co-regulatory networks. From the data derived for gene expression across r2–r5, several established regulatory networks have been correctly identified. *Gata2 *and *Gata3 *are expressed in the facial branchiomotor (FBM) of r4 and are known targets of Hoxb1 activity. Here *Gata2*/*3 *show similar microarray profiles to *Hoxb1 *and present in *Hoxb1*-derived clusters (Figure 5C, green arrow, and data not shown). This type of association can also be observed for the r3/5 Erg2-driven network where the known downstream targets *Epha4*/*7 *and *Nab1*/*2 *mimic the 'selector' gene profile. Together, the correct detection of known patterning genes as well as some of their downstream effectors suggests that this dataset is a faithful representation of gene expression in the segmented E9.5 hindbrain and can be used to confidently predict new network components.

### R4-specific retinoic acid signalling mechanisms

Assuming that Cyp1B1 is performing the same role in the ventral r4 domain as was described for chick Cyp1B1 (that is, synthesizing low levels of RA [70]), multiple functions can be envisioned. These include the production of low levels of RA to participate in an autoregulatory loop with Hoxb1 as well as potentially also being required to regulate the fate of the motor neurons born in the ventral r4 domain, to which its expression is restricted later in development (data not shown). To gain insight into these events, we are currently analysing the consequences of loss of Cyp1B1 function in r4. The role of continued RA expression in the regulation of cell fate has been previously recorded where RA released from cells within the ventral neural tube is required for the appropriate differentiation of motor neurons in that region [73].

### Other potential insights

The comparative lack of other identified transcription factors that respect rhombomere boundaries perhaps gives an insight into the nature of the regulatory network downstream of Hox. These observations imply that, generally, it is the Hox genes themselves that recognise target gene promoters and induce effectors rather than activating a second layer of transcription factors that subsequently induce cellular effectors (for example, cell adhesion molecules). There are exceptions to this, particularly with respect to neuronal populations within rhombomeres. In r4, we have identified and validated the expression of several transcription factors in the progenitors of the facial motor nuclei. These include known markers, such as Hoxb1, Nkx2.2, Gata2, Phox2b, Isl1, Neurod4 and Ebf1, but also other regulatory proteins identified here, such as bHLHb5 and Lmo1. The enriched expression of Lmo1 in r4 raises the intriguing possibility that it may have a role in regulating motor neuron identity.

Other genes validated in this screen are suggestive of unexplored signalling mechanisms operating during hindbrain patterning and the specification of cranial motor neurons. Tubby, a poorly characterised G protein coupled receptor, is enriched in both the trigeminal and facial motor regions, as is RGS4, a potent regulator of G protein signalling (Figure 3I',J').

### Segmental motifs and rhombomere relationships

To facilitate the interpretation of the gene expression dataset across the hindbrain, we have represented all of the observed profiles schematically (Figure 7). Our findings demonstrate that virtually all of the different combinations of gene expression profiles are present but that r2/4, r3/5 or individual rhombomeres dominate. This is consistent with previous findings about the molecular architecture and cellular behaviour of the segmental hindbrain. The diversity of the segmental expression patterns is also suggestive of as yet undescribed relationships within the developing hindbrain. Our data may provide an insight into some of these processes.

To get a 'global' quantitative measure of the relationship between rhombomeres at E9.5, we used hierarchal clustering across the 'changing and reliable' set of genes (Additional file 2A). Summation of existing evidence demonstrates that r3 and r5 are molecularly similar. However, these observations are based upon the expression of a relatively small number of key patterning genes (that is, *Egr2*, *Epha4*, *Nab1*/*2*, and so on). We applied hierarchical clustering to determine the magnitude to which these segments were molecularly similar or different; for example, to distinguish whether r3 and r5 differed principally by the expression of a few key selector and effector genes or whether the differences in 'selector' profiles were expanded to vastly different r3/5 identities. Our findings (Figure 8) demonstrate that there was a wide-ranging difference in the transcriptional profile of the odd- and even-numbered rhombomeres. As expected from regionally restricted expression of the *Egr2 *selector gene, we found that r3 and r5 displayed the highest level of relatedness across the 1,381 genes tested. These data give the first molecular readout of the depth of rhombomere relatedness and suggest that the selector profile of each rhombomere is further reiterated, via a complement of effector genes, to give wholly different segmental identities and neuronal territories.

### How do Hox genes pattern the hindbrain?

Rhombomeres are a transcriptionally diverse field of cells that express a myriad of transcriptional effectors in both specific neuronal subtypes (for example, in the motor neurons or interneurons) or across the whole field. Our data provide strong support to a model whereby Hox genes exert their effects via broad spectra of downstream targets. Given the multitude of neuronal cell types emerging from developing hindbrain, the challenge remains to decode and interpret the role of each potential effector with a distinction between those that impact on the property of the entire rhombomere at E9.5 and those that are confined to specific cell types within a rhombomere.

These data begin to give a picture of the complexity of transcriptional regulation that underlies the acquisition of cellular identity by hindbrain neuronal populations. For example, if we consider just the facial motor nuclei that emerges from r4, then the data provided here illuminate some of the transcriptional patterning events that may lead to its acquisition of the fine properties required to mark it as 'different' from the adjacent cranial motor nuclei. In addition to Gata2 and Gata3, several other genes are uniquely expressed or enriched in the r4 motor neuron progenitor domain, including *bHLHb5*, *Lmo1*, *Cyp1B1 *(see above), *Aldo3c *and *Gpx3*. Whilst the presence of nuclei-specific transcription factors may have readily interpretable effects on neuronal identity, it is more difficult to assess the role of Aldo3c, which catalyses the reaction of glycerine phosphate + D-glyceraldehyde 3-phosphate to give D-fructose 1,6-bisphosphate, and Gpx3, which catalyses the reaction 2 glutathione + H_2_O_2 _to give oxidized glutathione + H_2_O, in defining the observed cellular identity and behaviour. However, it is possible that enzymes such as these interact with as yet unidentified signalling pathways important for the acquisition of cell identity (for example, [74]). To this end, we are currently examining the potential functional roles of a number of the candidates identified here in patterning of the segmented hindbrain.

### Hox genes can regulate the level and not just the presence or absence of a gene in a tissue: 'horizontal' versus 'vertical' regulation

Where Hox genes function to modulate the fate of a given morphological module (for example, vertebral patterning), Cobb and Duboule [52] have proposed that Hox genes principally operate through a 'horizontal' regulatory strategy. Here it is proposed that morphological output may be the result of subtle variations to common genetic determinants rather than the initiation of distinct pathways. This is in contrast to the 'vertical' regulatory strategy where Hox genes drive the decision process leading to the emergence of a specific morphological structure.

Whilst the extent to which the horizontal and vertical regulatory strategies apply in any given situation, it is evident that in the segmental hindbrain, there is both modulation of gene expression level across the region as well as rhombomere-specific regulation. For example, *Mcm6*, *Hmgcs1*, *Nef3*, *Zfp313*, *Mtm1*, *Igfbp4*, *Ina*, *Pvrl3*, *Stmn2 *and many others are expressed in each rhombomere (as defined by a 'present' call) but show significant variation in their expression levels. The observed variation in gene expression level may be a result of several contributing factors ranging from a difference in the maturation state of the rhombomeres to an intended threshold of activity that drives unique rhombomeric cell fates. There is also clear evidence for a more 'vertical' approach to gene regulation across the hindbrain. Many of the genes appear to have unique rhombomeric expression profiles. Evidence for this is seen most clearly in r4, which itself is patterned by the intrinsic expression of *Hoxb1*. Here, we have presented evidence for the r4-specific expression of several genes, including *Cyp1B1*, *Aldo3c *and *Gpx3*, suggesting that at least some entirely distinct genetic pathways are being activated. Conversely, we have also uncovered evidence for r4-specific suppression (for example, *Thbs1 *and *Lix1*; data not shown) of gene expression.

Together, these data suggest that the appropriate emergence of neuronal identity in the hindbrain is the cumulative result of rhombomere-specific gene induction and repression in the context of other subtly regulated gene expression levels. The interplay between these factors will make unravelling the Hox regulatory network a difficult task. Progress in this field is likely to come from the union of several inter-related approaches to determining regulatory networks. Genomic approaches can now readily define the entire pool of genes expressed in a target tissue. Where possible, these data can be further refined by looking at sets of direct targets of transcription factors using chromatin-immunoprecipitation techniques (for example, ChIP-Chip [75] or ChIP-Seq [76]). Using these processes in combination with other genetic and proteomic studies, it will be possible to describe networks at an ever higher resolution. To begin to address this issue, we have initiated a 'location analysis' screen for several of the Hox genes to try and determine the breadth of the set of direct targets.

However, to fully assess the balance between modulation of gene expression across the hindbrain and rhombomere-specific expression, the complete set of differentially expressed genes needs to be assessed by further confirmatory techniques (for example, quantitative PCR and *in situ *hybridisation).

### Array sensitivity

The analysis of the data generated here suggests that the predicted gene expression profiles are reflective of those observed *in situ*. However, at E9.5 each rhombomere is already composed of multiple different cell types distributed across the dorsoventral axis and exposed to specific dorsally or ventrally located signals, as well as also being in various states of division. Together, this generates a heterogenous neuronal population that is likely to be responding to the Hox cues in subtle and different ways, even within a single rhombomere. This suggests that many transcriptional responses to Hox patterning may not be recorded due to being 'diluted' and thus falling below the limits of sensitivity. Whilst this is a consideration, the methodology described here was capable of accurately reporting gene expression changes associated with pools of rhombomere-specific cells (for example, *Isl1 *and *Gata2 *in cranial motor neurons associated with r2 and r4, respectively). Nonetheless, to obtain a truly comprehensive list of Hox target genes, each individual cell type within a rhombomere should be assayed. Emerging microarray technologies promise to deliver a more comprehensive survey of gene expression from smaller amounts of starting materials (for example, [77]). Advances in these technologies will allow us to accurately profile the transcriptional profile of a single homogenous cell type patterned by Hox genes (for example, VII motor nuclei of r4) and thus to perhaps more accurately predict the Hox effectors involved in specifying an individual neuron.

## Conclusion

Since the development of whole genome approaches to surveying gene expression, several studies have now described a systematic approach to defining the cascade of Hox downstream targets. This raises the possibility that in the future a consensus may emerge of the core set of genes regulated by Hox proteins. However, the extent to which Hox genes are used throughout development with respect to tissue and time suggests that in each context Hox genes activate a unique repertoire of targets. Due to the influence of cofactors and other modifying proteins, the set of targets derived from a single Hox gene in one tissue may be wholly unrelated to those regulated by the same gene in another tissue. Comparative analysis of datasets such as that described with other related screens [78] will significantly help address this issue. Furthermore, there is currently a significant research effort to direct the differentiation of neural stems towards a defined identity (for example, [79]). Together with other similar studies [19], the dataset described here provides a comprehensive set of genes that can be used to validate the appropriate activation of genetic programs for establishment of neuronal cell type associated with hindbrain patterning (for example, Hoxb1-dependent patterning in r4).

## Abbreviations

ANOVA: analysis of variance; AP: anteroposterior; E: embryonic day; GO: Gene Ontology; PCA: principle components analysis; r: rhombomere; RA: Retinoic acid, SEM: standard error ot the mean.

## Competing interests

The authors declare that they have no competing interests.

## Authors' contributions

DC devised and performed the experiments and analysed the data and wrote the manuscript. LJW and FA performed experiments, US, EH and EB analyzed data, and AL funded and devised the experiments and wrote the article.

## Supplementary Material

Additional file 1 **The relationship within and between biological replicates (r2–r5, sets 1–3) investigated using PCA and hierarchical clustering**. **(A) **Following normalisation and the removal of non-expressed genes, the overall distribution of gene expression levels in each sample was checked with PCA. Biological replicates are represented by similarly coloured dots (r2 = red, r3 = purple, r4 = light blue, r5 = yellow). The black circles group replicates from the same sample to show that biological replicates are more related to each other than any other sample. **(B) **Hierarchical clustering on the same dataset as described above shows that the biological replicates are closely related to each other and confirm the findings of PCA. These metrics suggest that the dataset from the hindbrain samples are suitable for further statistical analysis. Each gene in the cluster is represented by a single box at the same level and relative expression values in each rhombomere are depicted by the actual colour. Blue = underrepresented; yellow = equivalent to; and red = enriched with respect to the median expression value of that gene in all samples. Click here for file

Additional file 2 **Summary of data processing**. **(A) **Prior to statistical determination of differentially expressed genes, the dataset was assessed for its suitability for analysis (Additional file 1). Of the 22,900 probe sets printed on the MOE430A GeneChip, 8,870 were defined as not expressed and 19,253 were classified as not changing their expression between rhombomeres within a twofold limit. Of the remaining 1,381 candidates (defined here as 'changing and reliable'), one way analysis of variance (ANOVA) with a *p *= 0.05 cutoff parsed 381 genes as being the most statistically significant. **(B) **The 381 genes were grouped by biological process and cellular component as defined by GO (Additional file 3). Click here for file

Additional file 3 **The set of genes differentially expressed between r2 and r5 at E9.5. **Each gene is represented by its unique Affymetrix identifier, gene symbol and GenBank ID. Normalised expression values in each rhombomere are listed along with the GO molecular function, biological process and cellular location. Click here for file
