# Supplementary figures and images for: Rhombomere-specific analysis reveals the repertoire of genetic cues expressed across the developing hindbrain
Source: Neural Dev. 2009 Feb 10;4:6. doi: 10.1186/1749-8104-4-6 (PMC2649922; doi:10.1186/1749-8104-4-6)

**A**

PCA: changing and reliable gene set (1381)

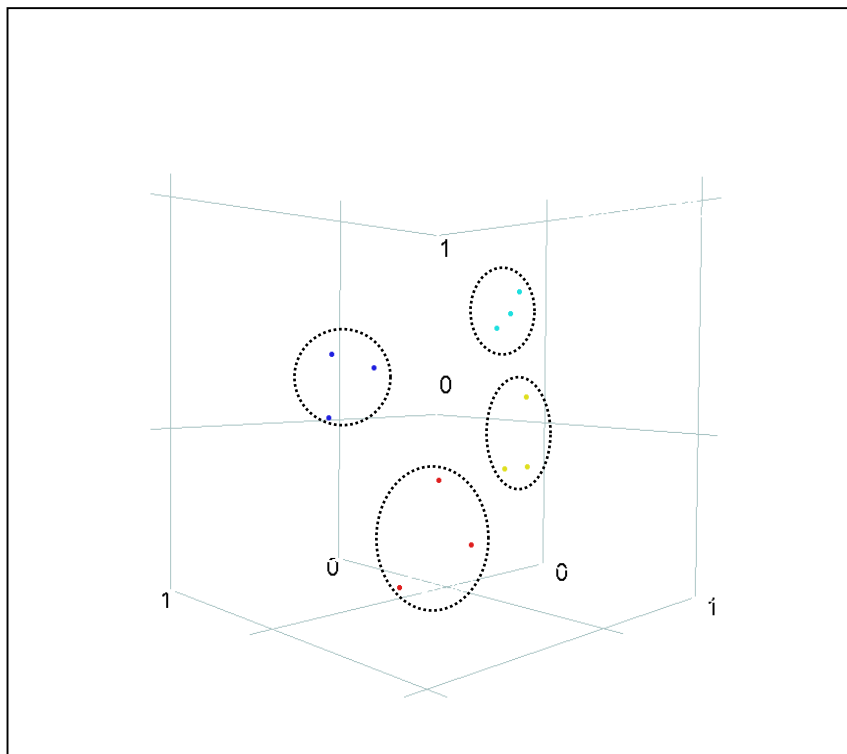

**B**

Hierarchical cluster: changing and reliable gene set (1381)

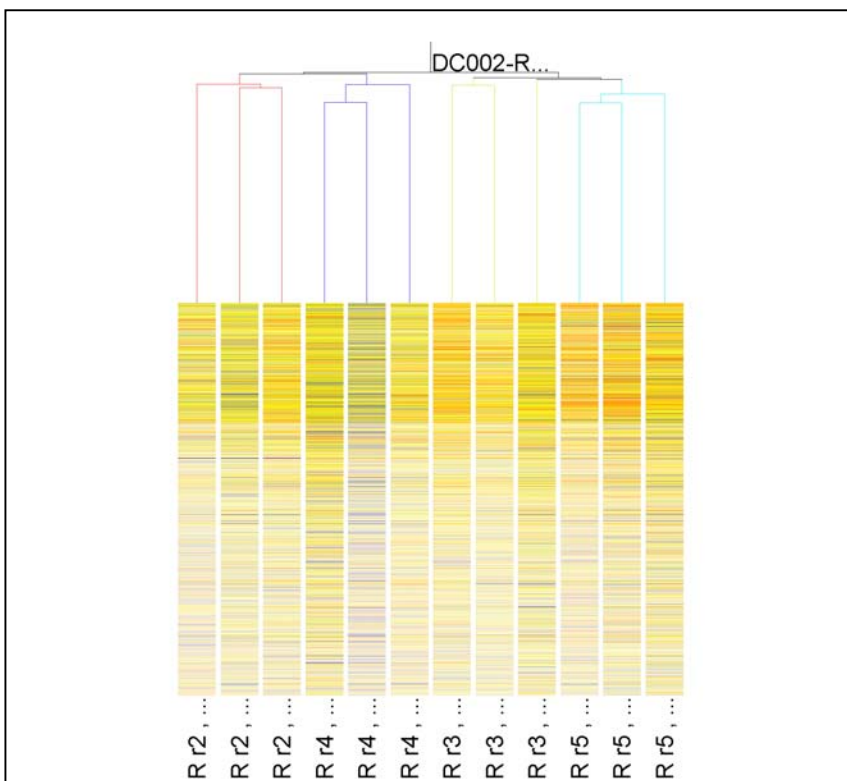

Supplement: Additional file 1 — The relationship within and between biological replicates (r2–r5, sets 1–3) investigated using PCA and hierarchical clustering. (A) Following normalisation and the removal of non-expressed genes, the overall distribution of gene expression levels in each sample was checked with PCA. Biological replicates are represented by similarly coloured dots (r2 = red, r3 = purple, r4 = light blue, r5 = yellow). The black circles group replicates from the same sample to show that biological replicates are more related to each other than any other sample. (B) Hierarchical clustering on the same dataset as described above shows that the biological replicates are closely related to each other and confirm the findings of PCA. These metrics suggest that the dataset from the hindbrain samples are suitable for further statistical analysis. Each gene in the cluster is represented by a single box at the same level and relative expression values in each rhombomere are depicted by the actual colour. Blue = underrepresented; yellow = equivalent to; and red = enriched with respect to the median expression value of that gene in all samples. [file 1749-8104-4-6-S1.pdf]

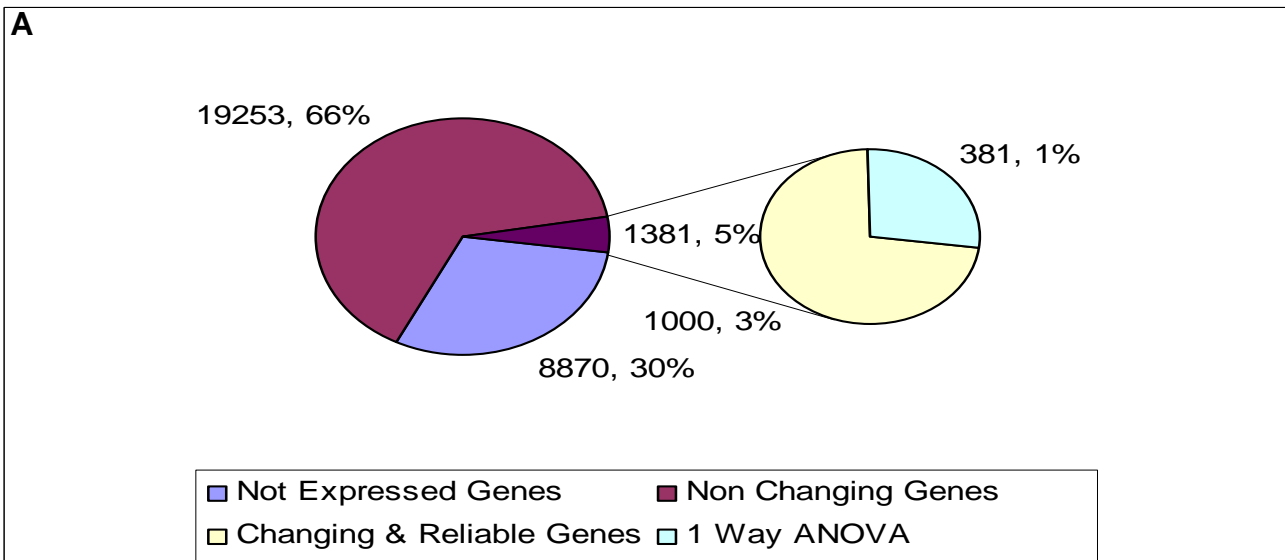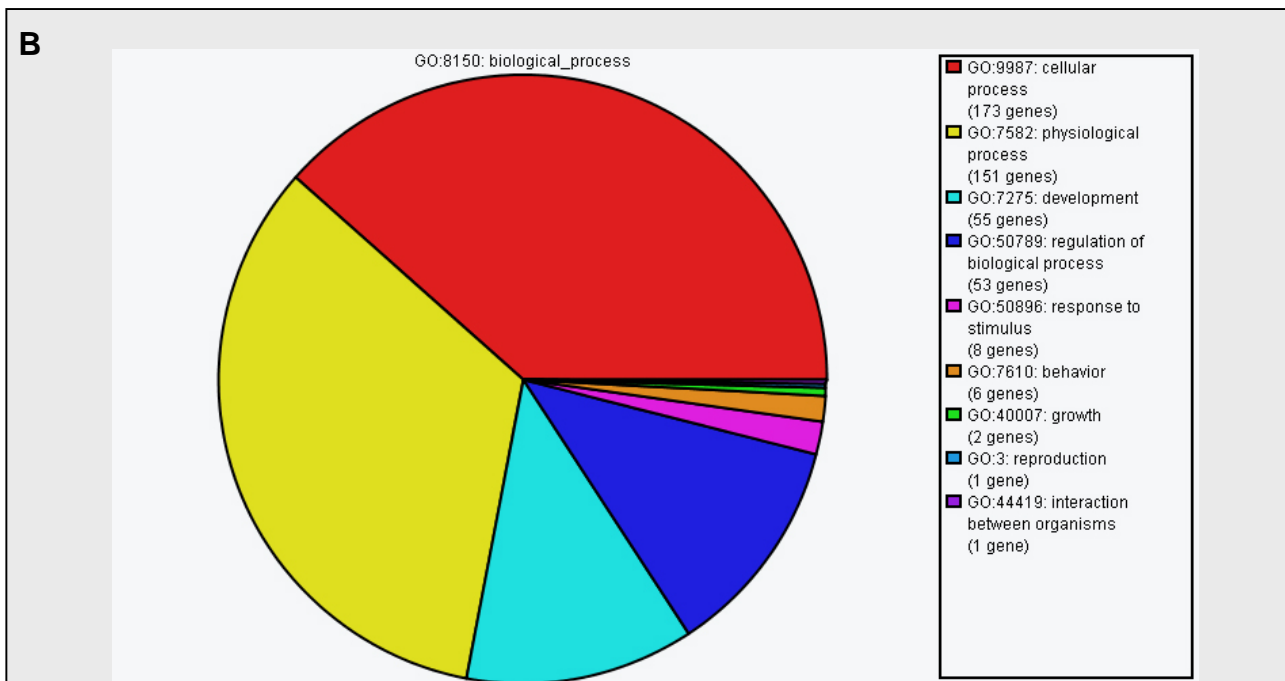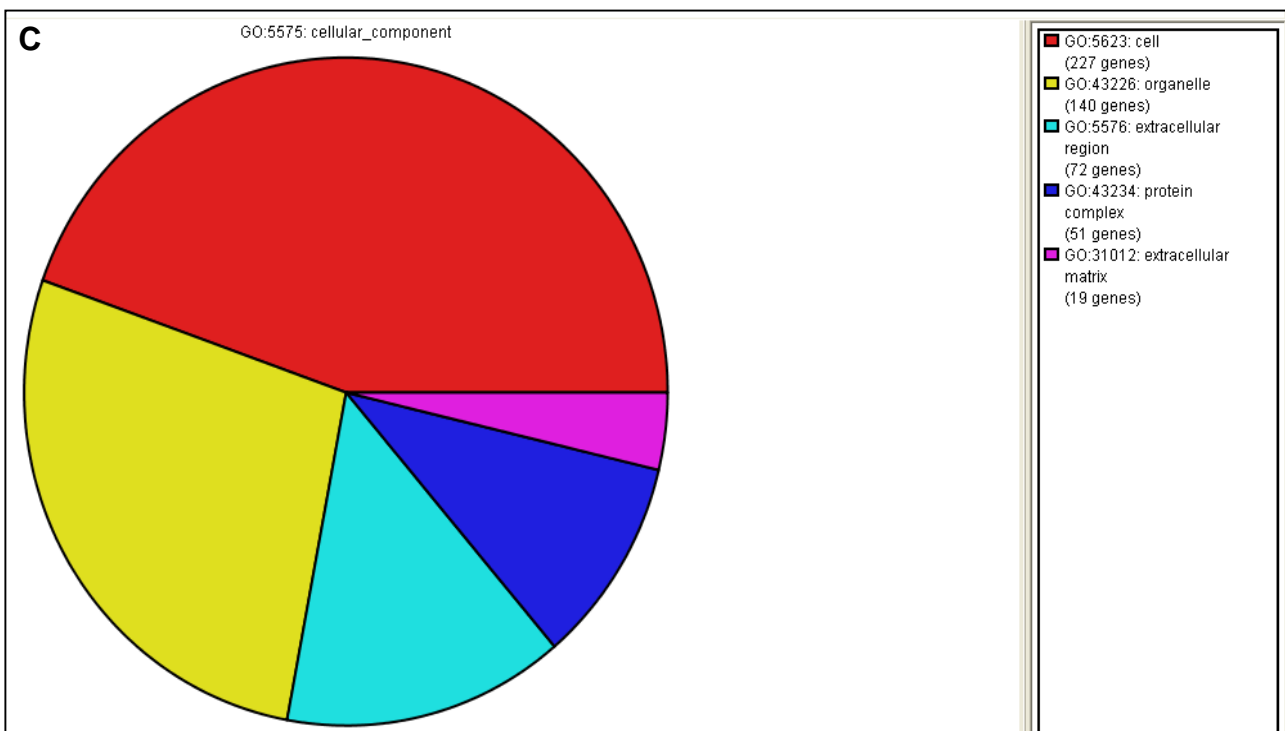

Supplementary Figure 2

Supplement: Additional file 2 — Summary of data processing. (A) Prior to statistical determination of differentially expressed genes, the dataset was assessed for its suitability for analysis (Additional file 1). Of the 22,900 probe sets printed on the MOE430A GeneChip, 8,870 were defined as not expressed and 19,253 were classified as not changing their expression between rhombomeres within a twofold limit. Of the remaining 1,381 candidates (defined here as 'changing and reliable'), one way analysis of variance (ANOVA) with a p = 0.05 cutoff parsed 381 genes as being the most statistically significant. (B) The 381 genes were grouped by biological process and cellular component as defined by GO (Additional file 3). [file 1749-8104-4-6-S2.pdf]
